# Evaluation of the Relationship Between the Invasive Front of Oral Squamous Cell Carcinoma and Clinicopathological Parameters

**DOI:** 10.30699/IJP.2021.520522.2541

**Published:** 2021-06-12

**Authors:** Nooshin Mohtasham, Narges Ghazi, Kazem Anvari, Farnaz Mohajertehran, Tahmine Organji, Mehdi Shahabinejad

**Affiliations:** 1 *Oral and Maxillofacial Diseases Research Center, Mashhad University of Medical Sciences, Mashhad, Iran*; 2 *Department of Oral and Maxillofacial Pathology, School of Dentistry, Mashhad University of Medical Sciences, Mashhad, Iran*; 3 Department of Radiotherapy Oncology and Cancer Research Center, Mashhad University of Medical Sciences, Mashhad, Iran

**Keywords:** Invasive front, Lymphocyte host response, Oral squamous cell carcinoma, Survival analysis

## Abstract

**Background & Objective:**

The present study investigated the relationship between invasive front (IF) of tumors and clinicopathological parameters including stage, grade, nodal involvement, lymphocytic host response (LHR), recurrence, overall survival (OS), and disease-free survival (DFS).

**Methods:**

A total of 87 oral squamous cell carcinoma (OSCC) biopsies were evaluated. Clinical stage, grading, nodal involvement, time of recurrence, OS, and DFS were assessed. The number of tumor budding cells in the IF was measured by two pathologists with an optic microscope. IF was graded to low risk (<5) and high risk (>5), according to the counting of tumor budding as a single cancer cell or cluster cells. Also, LHR was reported in the IF as mild, moderate, and severe.

**Results:**

IF was reported in 43.7% of patients as a low-risk group and 49.4% as a high-risk group. LHR was also mild in 31%, moderate in 25.3%, and severe in 43.7% of the patients. Most of the patients were in stage IV (31%) and grade 1 (60.9%). The high risk IF group had a significant statistical relationship with stage (*P*=0.001), grade (*P*=0.039), five years OS (*P*=0.03), five years DSF (*P*=0.01), and lymph node involvement (*P*=0.007). The relation between LHR and stage of disease was significant (*P*=0.034).

**Conclusion:**

Considering the essential role of histopathological reports in the treatment plan of patients and the relationship between IF and clinical parameters, IF evaluation in routine histopathological examinations, especially in the early stages of OSCC, seems to be necessary.

## Introduction

Oral squamous cell carcinoma (OSCC) accounts for 90% of oral neoplasm cases and is associated with high mortality and morbidity, with approximately 300,400 new cases, and 145,400 mortality cases around the world, annually (1, 2). The main treatment modalities of OSCC are surgery (removal of lesions, normal margin, and adjacent lymph nodes) and radiotherapy, either alone or in a combination (3). However, the current therapeutic options have not significantly increased the survival rate of OSCC patients, and recurrence and lymphatic metastasis are common causes of treatment failure (3). TNM classification of malignant tumors has been universally accepted as the prognostic marker for predicting OSCC. However, TNM classification does not always indicate a good prognosis at an early stage of oral cancers. Previous reports declared that 20% to 40% of oral cancers already have occult metastasis (4, 5). Also, due to the histopathological decision, “complete” resection in some OSCC patients in early stages leads to the recurrence of the disease. This point might be due to the remaining residual malignant cells with the potency to migrate and metastasize even after treatment (6). 

Differentiation of epithelial–mesenchymal transition (EMT) cells from stromal fibroblasts is performed by light microscopy. It is believed that the stem cells are resistant to therapy, and it might be proposed that cancer stem cells in the front zone of tumors migrate (7, 8). Invasive front (IF) is a place that the first tumor cells are released from the epithelium and invade the connective tissue. IF indicates two components: loss of adhesion and active invasive movement that can play an important role in the prognosis of the malignancy. Therefore, the IF is considered an important zone in the progression of tumors. This zone has an active invasion and crosstalk between tumor and stroma (9). The IF pattern is a method for tumor progression; if cancer cells have extensive spread infiltration from the EMT, it is considered as invasive tumor pattern and not the bulky pushing infiltration pattern (10-13). In recent years, the pattern of IF alone or as a part of the scoring system, could be indicative of the prognosis of locally recurrent tumors and the OS of the patients (14). Tumor budding is demarcated as the presence of single carcinoma cells or bulky pushing clusters of cells (below) 5 cells located at the IF of neoplastic epithelial tumors. 

However, tumor budding theory have been described before (15), its related pathology (16), presentation as a prognostic factor and tumor invasion pattern have been defined recently (14, 17-20). This theory is described as attenuation of the cellular adhesion as well as the presence of invasion at the IF zone and has been advanced to be closely associated with epithelial to mesenchymal transition (16, 21). There are various important patholo-gical factors during OSCC process. However, there are few studies on the prognostic role of the IF in OSCC cancer (22). In the present study, we aim to evaluate the prognostic role of IF considering the clinicopathological markers including histopathological grade, stage of the disease, lymphocyte host of response (LHR), and overall survival (OS) and disease-free survival (DFS) among OSCC patients.

## Materials and Methods

**Patients and Tumors**

This observational-retrospective study was conducted on 87 biopsy samples taken from the archive of Pathology Departments in Ghaem Hospital, Omid Hospital and Imam Reza Hospital, Mashhad University of Medical Sciences (MUMS), Mashhad, Iran. These patients were referred to Omid Hospital from 2005 to 2017, having their files documented. Demographic information of patients including age, sex, smoking and drug history were recorded. Ethics Committee of MUMS has approved the study protocol under Number of IR.mums.sd.rec.-1394.322. Available OSCC biopsy samples were include-ed from samples with adequacy to examine IF. Also, the other inclusion criteria were patients between 18 to 80 years old, who had not received any antitumor therapy during sampling, patients who didn’t have distant metastases and radical surgery and no tumor remnants after surgery, patients who had a complete record and follow-up of at least six months, samples with excisional biopsy and healthy margin around the tumor. Moreover, patients who were followed for less than six months, patients with distant metastasis, patients with stage IVb, c, and OSCC patients with lesions of vermilion, the base of the tongue, throat, and larynx were excluded.

**Follow-up**

The follow-up time (mean=75 months, range=6-144 months) was measured from the time of cancer diagnosis. Complete follow-up information, including disease recurrence and cause of death, were collected from the outpatient and the hospital charts, autopsy reports, and family physicians. Besides information on the node lymph involvement, degree of tumor grade, and stage of tumor based on the recorded information and recurrence and survival of patients were all collected via contacting every case using a checklist.

**Histopathology**

Formalin-fixed, paraffin-embedded biopsies were prepared according to standard procedures for hemat-oxylin-eosin (HE) staining. Two pathologists verified the diagnosis of OSCC and histopathologically evaluated the tumors in a blinded approach from clinicopathological parameters of studied samples. IF was graded according to the number of tumor buddings as a single cancer cell or a cluster, to low risk (<5) and high risk (>5) (23). Slides were scanned using 40× and then, 100× of magnification to select areas with the highest tumor buddings. Tumor buddings were counted using 400× magnification and the highest count area per slide was selected as the score of budding (24).

Therefore, LHR is evaluated at the tumor interface, and categorized into strong (>50%), intermediate (25%-50%), and weak (5%-25%) according to the presence of lymphocytes collection areas which were directly adjacent to the tumor-host interface (14, 25). 

**Statistical Analysis**

Data were statistically analyzed by SPSS 20 (SPSS Inc., Chicago, IL, USA). Chi-square analysis was performed to analyze the relationship between the IF and studied clinicopathological parameters including stage, grade, and lymph node involvement. Besides, the relation between LHR and stage, grade and node involvement were analyzed by the chi-square test. Kaplan–Meier survival analysis was performed, and three years and five years of OS and DFS were computed. The endpoints for survival analysis were the time of OSCC recurrence, the time of last follow-up or the time of death.

## Results

**Clinicopathological Findings**

**Fig. 1:**
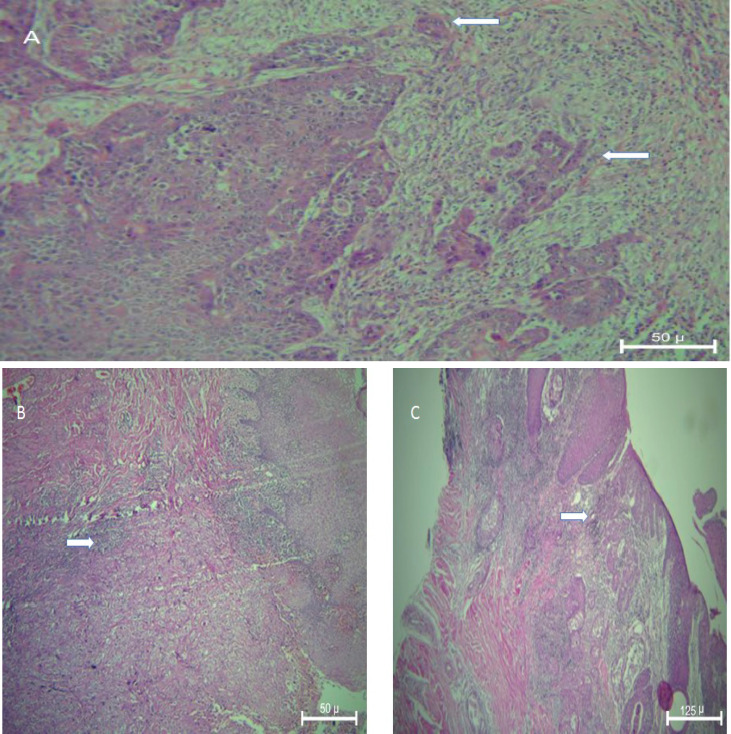
A: Low risk IF in early stage and Grade 1 SCC (Arrows) X100, B: High risk IF in advanced stage and Grade III (ARROWS) X100, C: High risk IF in advanced stage and Grade II(ARROWS) X40

**Fig. 2:**
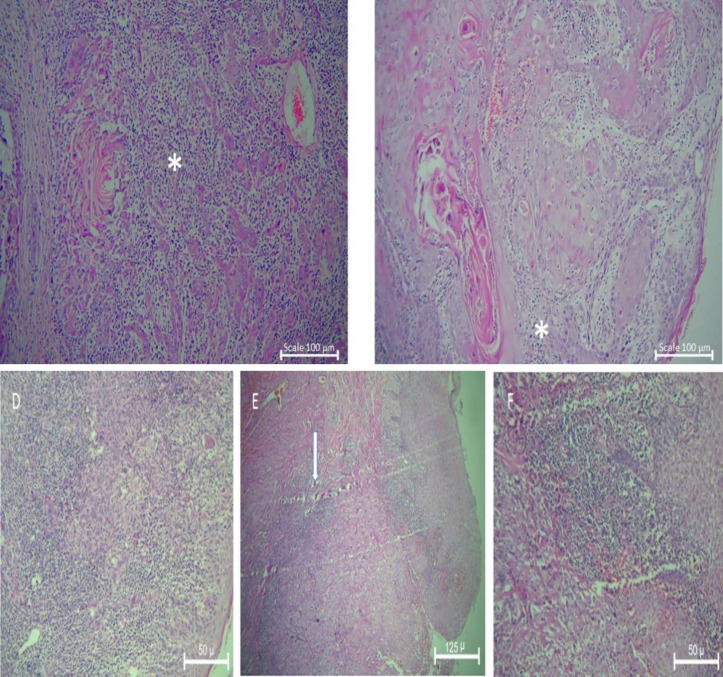
A: High-grade SCC with sever chronic inflammatory infiltration (asterisk). X100, B: Low-grade SCC with mild to moderate chronic inflammatory infiltration (asterisk). X100

In the present study, 87 biopsy samples of OSCC including 44 female and 43 male patients aging from 18 to 85 years were evaluated. Clinicopathological param-eters of studied biopsies are presented in Table 1. There were 44 and 43 biopsies in the low-risk and high-risk IF category, respectively. Most of the studied biopsies (43.75) had severe LHR and were in stage IV (31%). However, there was not any significant relationship bet-ween the distribution of different stages, histopathological grades, and different LHR (*P*>0.05). Most of the patients had negative lymph node involvement (58.6%). Inflam-matory response at low risk IF in early stage and grade I SCC with 100× magnification and in high risk IF in Grade II and III is presented in Figure 1 (A, B,C, respectively). The relation between IF and studied clinicopathological parameters was shown in Table 2. Statistical analysis showed that there was a significant relationship between the stage of the disease and different categories of IF (*P*=0.001). Most of the patients with low risk IF (38.6%) and most of them with high risk IF (46.5%) were in stage I and stage IV, respectively. There were 75% and 46.5% of studied patients in grade I with low risk IF and high risk IF, respectively. Figure 2 showed low risk IF in early stage and high LHR and high risk IF in advanced stage and mild to moderate LHR with 100× and 40× magnification. Besides, 20.5% and 41.9% of studied patients in grade II were categorized in low risk and high risk IF groups, respectively. The relation between histopathological grade and different IF groups was statistically significant (*P*=0.039, Table 2). Also, the relation between lymph node involvement and IF was significant (*P*=0.007, Table 2). Most of the patients with negative node involvement (55.8%) were in the low risk IF group. While most of the patients (72.70%) with positive lymph node involvement had high risk IF (*P*=0.07, Table 2). 

**Table 1:** Clinicopathological parameters of studied samples

Variable		Number (Percentage)
Invasive front	Low risk High risk	44 (50.6) 43 (49.4)
Lymphocytic host response	Mild Moderate Severe	27 (31) 22 (25.3) 38 (43.7)
Histological stage	I II III IV	22 (25.3) 19 (21.8) 19 (21.8) 27 (31)
Histological grade	1 2 3 4	53 (60.9) 27(31) 5(5.7) 2(2.3)
Nodal involvement	Negative Positive	51 (58.6) 36 (41.6)

**Table 2:** Relation of invasive fronts with studied clinicopathological parameters

Variable		Invasive front		P-value (χ2)
		Low risk	High risk	
Histological stage	Stage I Stage II Stage III Stage IV	17 (38.6) 13 (29.5) 7 (15.9) 7 (15.9)	5 (11.6) 6 (14) 12 (27.9) 20 (46.5)	0.001 (16.69)
Histological grade	Grade 1 Grade 2 Grade 3 Grade 4	33 (75) 9 (20.5) 2 (4.5) 0	20 (46.5) 18 (41.9) 3 (7) 2 (4.7)	0.039 (8.37)
Nodal involvement	Negative Positive	32 (27.3) 12 (72.70)	19 (44.2) 24 (55.8)	0.007 (7.30)

D: Low risk IF in early stage and High LHR (X100), E: High risk IF in advanced stage and mild to moderate LHR (arrow) ( X40 ), F: X100 magnification of figure

The relationship between LHR and studied clinicopathological parameters is shown in Table 3. Most of the studied samples (74%) with mild LHR were in stage III and stage IV. Also, most of the samples with moderate (40.9%) and severe (36.8%) LHR were in stage IV and stage I, respectively. There was a significant relationship between the stage of disease and LHR category (*P*=0.03, Table 3). The relationship between histopathological grade and LHR showed that most of the studied samples into three different categories of LHR were in grade I. There was not any significant difference between the grade of disease and LHR (*P*=0.15, Table 3). There were 59.3%, 36.4% and 31.6% of samples with positive lymph node involvement and mild, moderate, and severe LHR, respectively. Moreover, the relation between lymph node response and LHR was not significant (*P*=0.07, Table 3).

**Survival Findings**

**Fig. 3:**
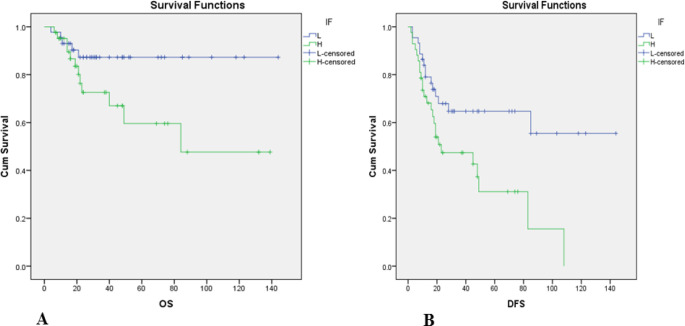
Relation of overall survival (OS) (A) and disease free survival (DFS) on invasive front (IF), L: Low risk, H: High risk

**Fig. 4:**
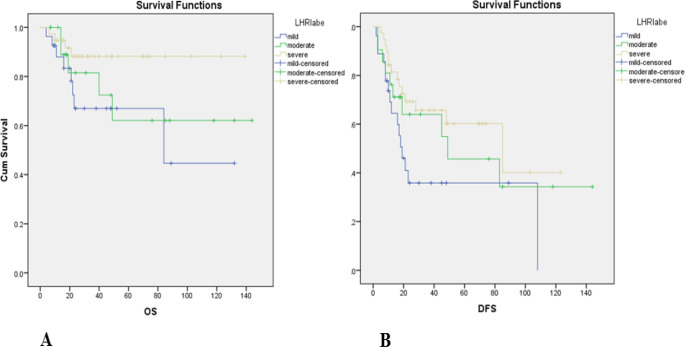
Relation of overall survival (OS) (A) and disease free survival (DFS) on lymphocyte host response (LHR)

Evaluation of the relation between the IF and five years OS and DFS are depicted in Figure 3. Analysis of five years OS was significantly different in high risk IF (59.6%±10.7%) and low risk IF (87.3%±5.4%) patients (*P*=0.03, Table 4). Also, three years OS was 72.6%±8% in high risk IF and 87.3%±4.4% in low risk IF patients. Besides, DFS was significantly different in high risk IF (72.8%±6%) from low risk IF (87.4%±3.4%) patients (*P*=0.03, Table 4). Three years DFS was 47.4%±8.3% in high risk IF and 64.7%±7.8% in low risk IF patients. Evaluation of the relation between LHR, five years OS and DFS are shown in Figure 4. Five years OS was not significantly different in mild (358%±10.3%), moderate (45.7%±13.6%), and severe LHR (60.2%±9.1%) patients (*P*=0.08, Table 4). Three years OS was 87.3%±4.4% in mild LHR, 64%±11.2% in moderate LHR, and 65.7%±8.2% in severe LHR patients. DFS was not significantly different between patients with mild (67%±10.5%), moderate (62.1%±14.20%), and severe (88.2%±5.6%) LHR (*P*=0.10, Table 4). Three years DFS was 67%±10.5% in mild LHR, 81.5%±9.8% in moderate LHR, and 88.2%±5.6% in severe LHR patients. 

**Table 3:** Relationship between lymphocyte host responses with studied clinicopathological parameters

Variable		Lymphocytic host response			P-value (χ2)
		Mild	Moderate	Severe	
Histological stage	Stage I Stage II Stage III Stage IV	2 (7.4) 5 (18.5) 10 (37) 10 (37)	6 (27.3) 3 (13.6) 4 (18.2) 9 (40.9)	14 (36.8) 11 (28.9) 5 (13.2) 8 (21.1)	0.034 (13.6)
Histological grade	Grade 1 Grade 2 Grade 3 Grade 4	12 (44.6) 11 (40.7) 2 (7.4) …	13 (59.1) 7 (31.8) 2 (9.1) …	28 (37.8) 9 (23.7) 1 (2.6) …	0.15 (9.36)
Nodal involvement	Negative Positive	11 (40.7) 16 (59.3)	14 (36.8) 8 (36.4)	26 (68.4) 12 (31.6)	0.071 (5.29)

**Table 4:** Evaluation of overall survival and disease free survival on invasive front and lymphocytic host response (LHR)

Variable		Overall survival Mean±SD	Disease free survival Mean±SD
Invasive front	High risk Low risk P (long rank chi)	59.6±10.7 87.3±5.4 0.03 (4.29)	31.1±1.5 64.7±7.8 0.01 (6.67)
LHR	Mild Moderate Severe P (long rank chi)	35.8 ±10.3 45.7±13.6 60.2±9.1 0.08 (4.93)	67±10.5 62.1±14.2 88.2±5.6 0.10 (4.45)

## Discussion

Previous studies have reported that tumor budding is associated with poor prognosis and metastasis of lymph nodes in several types of carcinomas (10). Moreover, tumor buddings more than five have been declared to be associated with a poor prognosis (24, 26, 27). Cancer metastasis is described as invasion of cancerous cells to the stroma and vessels, their colonization, and their proliferation in the lymph nodes (28, 29). The association between EMT and tumor have been previously described (24). Tumor budding could be considered as an early step in cancer invasion (30), and patients with tumor buddings are at risk of cancer progression. In this study, patients were categorized into high risk IF and low risk IF groups according to their tumor budding status. 

Our study objective was to evaluate the association between IF and clinicopathological parameters includ-ing the stage of the disease, histopathological grade, and lymph node involvement. We investigated OS and DFS based on IF grading. The results of this study showed that there was a significant direct relationship between the stage of disease and IF. As most of the patients in higher stages of disease (IV and III) were in the high-risk group of IF and patients in stage I and II were in the low risk IF group. A significant relation was shown between OSCC grade and IF; 75% of patients in the low-risk IF category and 41.9% of patients in the high risk IF category were in grade I and grade II, respectively. Patients in the high-risk IF group had higher positive node involvements compared to patients with low risk IF. 

Studies aiming to search the prognostic evidence in the early stage of oral cancers should identify the pa-tients who are at risk of adverse outcomes and aggr-essive treatment and the patients who have increased chances of a favorable outcome. The local surgical treatment alone should be adequate for the latter group (31). However, as previously described, TNM classification in the early stage of oral cancers is not successful in differentiating these two groups of patients (32). 

Similar to our results, Almangush *et al.* (27) found that tumor buddings with more than five clusters at the IF of the tumor were associated with poor prognosis after searching for better prognostic approaches compared to TNM evidence in the early stage of oral tongue SCC. In another study (24), Statistical analysis revealed that tumor budding was significantly ass-ociated with tumor size, differentiation, clinical stage, and lymph node metastasis. Furthermore, they found that tumor budding at the IF is correlated with reduced OS (24). Previous reports have concluded that a high grade of tumor budding and adjacent tissue at the IF can serve as useful predictors of delayed neck metastasis in the early stage of tongue cancer (33). This study did not report number of cells in tumor buddings and OS in five years follow-up. Our study investigated the survival analysis (Kaplan–Meier) and showed that five years OS in high risk and low risk IF was 59.6%±10.7% and 87.3%±5.4%, respectively. There-fore, patients with high-risk IF had a weaker prognosis compared to patients with low risk IF. DFS analysis showed that DFS in high risk and low risk IF patients was 31.1%±9.5 and 64.7%±7.8%, respectively. Given that, number of tumor budding cells more than 5 could be proposed as the prognostic factor of local recurrence. 

IF of the tumors is a region that effects the biological nature of tumors. In this region, there are various factors related to epithelium and mesenchyme cells that definitely affect each other. The most important factors related to epithelium in IF region is the attachment of epithelial cells which is usually provided by E-Cadherin molecules and their adapter (β-Catenins). Any changes in these molecules leads to displacement, dispersion, movement of tumoral cells and capability of epithelial cells to invade and metas-tasize to adjacent tissues, lymph nodes and eventually distant tissues. The other adhesion molecules are CD44 and Selectins which not only have a role in cell adhe-sion, but also affect cellular differentiation. Downregu-lation of these molecules contributes to lack of inter-cellular connectivity and decremention of tumoral cells. Some EMT factors in IF region which are effective in tumorigenesis and invasion processes, include collagen type IV, Laminin, Fibronectin, MMPs, miRNA, Oncofetal antigens, Podoplanin and also Glucose-transporter-1 that saves tumor cells from hypoxia (34). According to several studies, the biological margin of the tumor in IF region may not match the surgical margin of the SCC. Biological factors related to epithelium and EMT in this area may play important roles in the disease prognosis and response to treatment (20).

Recently, consideration of LHR response to the IF as a prognostic predictor of oral cancer metastasis has been demarcated (35). LHR has been reported to be associated with a favorable prognosis (14, 36, 37, 38, 39). In this investigation, concentrating on the tissue surrounding the tumor, we evaluated the types of adjacent tissue related to LHR categories at the tumor IF and the association between its presence and clinicopathological parameters. In this regard, a significant inverse association between stage of disease and LHR categories was observed. Most frequent stages of disease in patients with mild (37%), moderate (40.9%), and severe LHR (36.8%) were related to stage III, IV, and I, respectively. However, LHR did not have any significant relation with the grade, lymph node involvement, OS, and DFS analysis. This might be due to the number of studied samples. Similar to our study results, Brandwein *et al.* (14) found that there was a significant inverse relation between LHR and stage of the disease but there was not any significant relation between OS and local recurrence. 

Evaluating every available document from all of the included OSCC patients and contacting all of them in order to investigate the cause of death and any recurrence of the disease, could be considered as the advantage of this study. Yet, our most important limitations in conducting this research project were inadequacy of patients' files, lack of documents in some cases in terms of follow-up and referral to the related hospital, and lack of access to the phone numbers of all patients. Therefore, we had to exclude a portion of the studied sample. Due to the small sample size, we could assess the prognosis of IF high-risk patients only in the early stage. Furthermore, we found that due to the higher stage of the disease, patients with high risk IF had a poor prognosis.

## Conclusion

To conclude, we report the association of high tumor-budding index or high-risk IF category with poor prognosis in patients with OSCC. Tumor budding was significantly associated with stage, grade, and involvement of node metastasis. Due to the association between five years OS and DFS with invasive front, it is concluded that scoring of buds in OSCC patients could help to discriminate invasive tumors that are prone to relapse. Due to the importance of OSCC as the most common oral cancer, the results of this study could be applicable for further studies on the IF into predicting OS and DFS for patient evaluation. Therefore, evaluation of invasive front in usual histopathological examinations, especially in the early stages of OSCC, seems to be necessary.
